# Integration of multiple terminology bases: a multi-view alignment method using the hierarchical structure

**DOI:** 10.1093/bioinformatics/btad689

**Published:** 2023-11-16

**Authors:** Peihong Hu, Qi Ye, Weiyan Zhang, Jingping Liu, Tong Ruan

**Affiliations:** School of Information Science and Engineering, East China University of Science and Technology, Shanghai 200237, China; School of Information Science and Engineering, East China University of Science and Technology, Shanghai 200237, China; School of Information Science and Engineering, East China University of Science and Technology, Shanghai 200237, China; School of Information Science and Engineering, East China University of Science and Technology, Shanghai 200237, China; School of Information Science and Engineering, East China University of Science and Technology, Shanghai 200237, China

## Abstract

**Motivation:**

In the medical field, multiple terminology bases coexist across different institutions and contexts, often resulting in the presence of redundant terms. The identification of overlapping terms among these bases holds significant potential for harmonizing multiple standards and establishing unified framework, which enhances user access to comprehensive and well-structured medical information. However, the majority of terminology bases exhibit differences not only in semantic aspects but also in the hierarchy of their classification systems. The conventional approaches that rely on neighborhood-based methods such as GCN may introduce errors due to the presence of different superordinate and subordinate terms. Therefore, it is imperative to explore novel methods to tackle this structural challenge.

**Results:**

To address this heterogeneity issue, this paper proposes a multi-view alignment approach that incorporates the hierarchical structure of terminologies. We utilize BERT-based model to capture the recursive relationships among different levels of hierarchy and consider the interaction information of name, neighbors, and hierarchy between different terminologies. We test our method on mapping files of three medical open terminologies, and the experimental results demonstrate that our method outperforms baseline methods in terms of Hits@1 and Hits@10 metrics by 2%.

**Availability and implementation:**

The source code will be available at https://github.com/Ulricab/Bert-Path upon publication.

## 1 Introduction

Medical terminology bases, as ICD-10 (Steindel 2010), ICD-11 (Harrison *et al.* 2021), and SNOMED-CT (Donnelly 2006), encompass an extensive collection of medical concepts, including their definitions, relationships, synonyms, and classifications. These bases are essential for standardizing medical terminology (Luo *et al.* 2019) and facilitating information retrieval. Furthermore, several studies (Michalopoulos *et al.* 2021) have explored the potential of utilizing terminology bases as domain-specific knowledge for language models to improve the accuracy of various tasks. However, relying solely on a single terminology base may be insufficient for retrieving comprehensive and innovative medical data. Consequently, there arises a need to integrate multiple terminology bases, which involves identifying related concepts across different medical systems. Interestingly, the task of integrating multiple terminologies exhibits a striking resemblance to the harmonization task encountered in domain ontologies. The structure of these medical terminology bases closely resembles that of medical domain ontologies and has even been considered as medical ontologies in certain research studies (Castell-Díaz *et al.* 2023). This may indicate that terminologies and ontologies encounter similar structural challenges and a robust integration approach also holds significant importance for aligning and constructing domain ontologies.

In the process of integrating multiple terminology bases, terminology alignment is a crucial step that aims to identify equivalent entities across different terminology bases. We adopt the definition of terminology base as a specific type of knowledge graph, as proposed by some studies (Zhang *et al.* 2020), and use entity alignment methods to solve the terminology alignment problem. The mainstream approach to entity alignment currently involves mining information on both the structural and semantic aspects of entities. Structural information is represented by models such as Graph Convolutional Networks (GCNs) (Wang *et al.* 2018), including HGCN (Gao *et al.* 2022), GAT (Xie *et al.* 2020), and RDGCN (Wu *et al.* 2019), which have been widely used for graph structure aggregation. Regarding semantic features, the BERT-based methods (Devlin *et al.* 2019) have emerged as the mainstream approach due to their capability to capture contextual information. Specifically, in the biomedical field, domain-specific models like PubMedBERT (Xie *et al.* 2022), trained on abstracts from biomedical literature, show great potential in capturing biomedical semantics. In addition to these methods, some approaches have attempted to leverage both structural and semantic feature for entity alignment, as HMAN (Yang *et al.* 2019) and BERT-Int (Tang *et al.* 2020).

**Figure 1.**
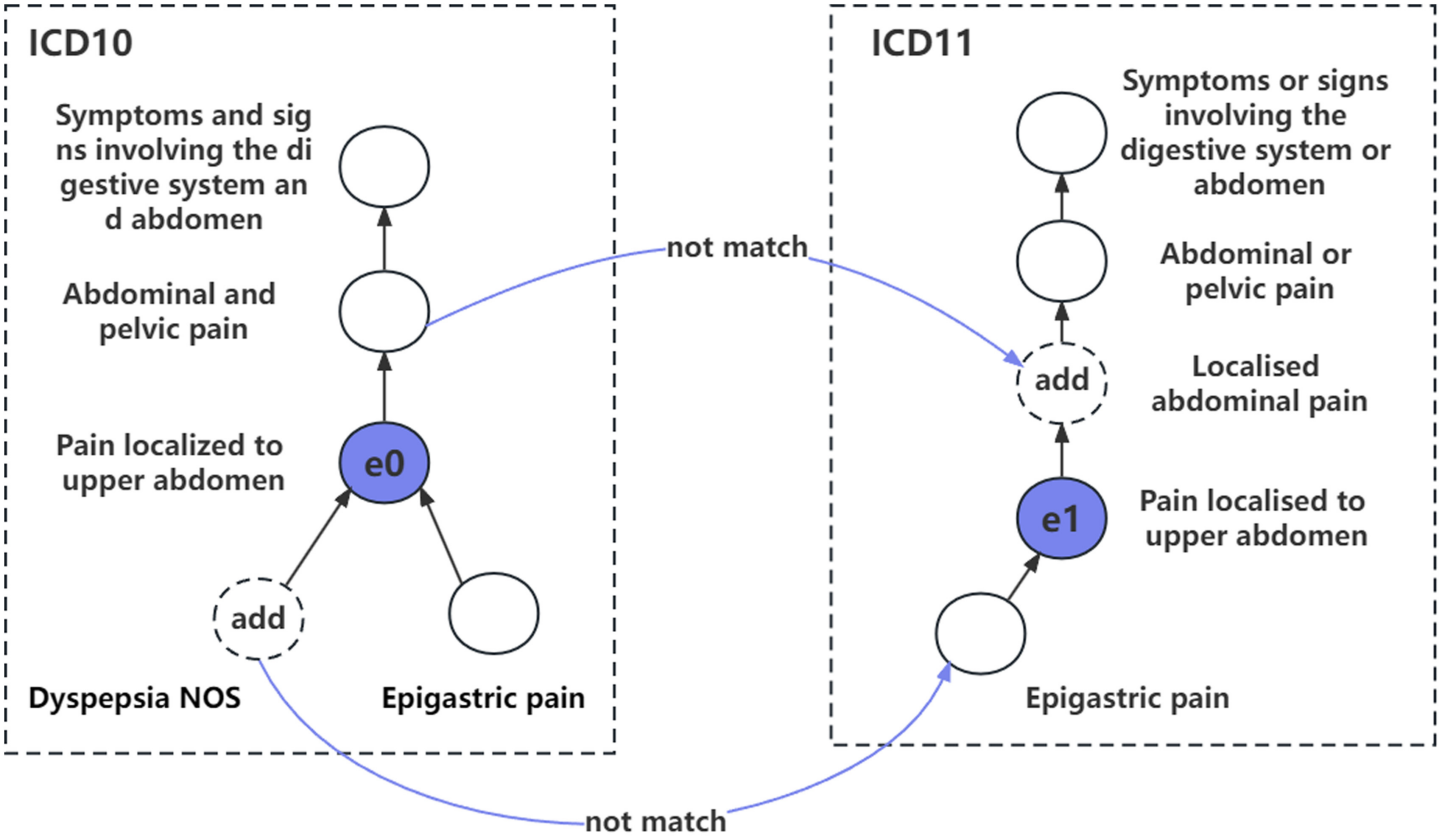
Structural differences between ICD-10 and ICD-11. The e0 and e1 represent aligned seeds, and the dashed box “add” represents newly added terms between the two systems, resulting in differences in neighborhood.

However, applying existing methods to the medical terminology alignment can be challenging, as the neighborhood structures of entities in different medical systems can vary significantly and change frequently. This can introduce inconsistent information and result in alignment errors, as shown in Fig. 1. Consequently, the same term may be assigned to a new category, resulting in mismatches in the parent nodes between the two versions. For example, the term “*Pain localized to upper abdomen*” is classified under the category of “*Abdominal and pelvic pain*” in ICD-10, while it belongs to the category of “*Localized abdominal pain*” in ICD-11. Such inconsistencies in neighborhood structure of the two versions of ICD may pose significant challenges for their alignment.

To address the problem of inconsistencies in neighborhood between terminologies, it is crucial to incorporate additional information that can help compensate for these differences. We propose a multi-view approach that leverages multiple sources of information to align medical terms, including their names, neighborhood, and hierarchical structure. By incorporating these different views of medical terms, our approach can capture diverse features that are complementary to each other, leading to more accurate results. Specifically, our approach involves incorporating interactive scores of the hierarchical paths into the alignment process, which reduces errors caused by differing levels between terminologies. For example, consider the case of “*Pain localized to upper abdomen*” mentioned earlier. Although the parent nodes of the terminologies are different, they shared a common ancestor node of “*Abdominal and pelvic pain.*” By using hierarchical features, we are able to reduce errors caused by ambiguous neighborhood information, resulting in improved recall and precision. In the final output, we apply gating mechanisms to assign different weights to the feature vectors, resulting in the final scores.

We validate our proposed alignment method using three publicly available medical terminology mapping files, including ICD10-ICD11, ICD9CM3-SNOMED, and SNOMED-ICD10. Our results indicate that our proposed method outperforms traditional methods on all three datasets. Specifically, by incorporating hierarchical information into the alignment process, our method achieved about 2% improvement in Hit@1 compared to the previous BERT-Int method for the ICD10-ICD11 terminology bases.

To sum up, the main contributions of our work can be summarized as follows:

- We have discovered a novel hierarchical feature in the terminology base that has a significant impact on the final alignment accuracy.
- We propose a multi-view model that integrates hierarchical, neighborhood, and semantic information to facilitate the process of term alignment.
- The experimental results demonstrate that the proposed model outperforms other baseline methods.

The rest of this paper is structured as follows. Section 2 presents the definition of our task, while Section 3 details our proposed model. Experimental results are reported in Section 4, and we conclude the paper in Section 5.

## 2 Method overview

In this section, we present an exposition of the fundamental concepts that underpin our study, and then formally define the terminology alignment problems. Lastly, we introduce our framework for the proposed method.

Definition 1.  (Biomedical Terminology Base.) A biomedical terminology base *G* is a directed acyclic graph. Given the *G*, we can define three types of triples, as follows: $\left(e_{i},h_{ij},e_{j}\right)$ indicates that concept *e_i_* has parent concept *e_j_*. $\left(e_{i},r_{ij},e_{j}\right)$ indicates that concept *e_i_* has an attribute relationship *r_ij_* with *e_j_*. (*e_i_*, *s_ij_*, *e_j_*) indicates that node *e_i_* is a synonym of concept *e_j_*. In addition, we define $N(e)={\left\{(e,e_{i})\right\}}_{i=1}^{\left|N(e)\right|}$ to represent the set of all neighbors of entity *e*. Similarly, we use $P(e)={\left\{(e_{i},h_{ij},e_{j})|e_{k}=e\right\}}_{i=1}^{\left|t\right|}$ to represent a hierarchical path of length *t* for entity *e*. Here, *k* represents the position of seed entity *e* within the path, *e*_1_ denotes the beginning concept of the path, and *e_t_* represents the end of the path.

Problem 1.  (Terminology Alignment.) Given two terminology bases *G* and G′, and a set of prealigned entity pairs I=(e∼e′), terminology alignment aims to learn a ranking function f:E×E′→R to calculate a similarity score between two entities. Based on this score, we rank the correctly aligned entity e′ as high as possible among all entities in E′, when queried for any entity *e*. The ultimate objective is to identify the entity e′ with the highest score as the most suitable alignment for the entity *e*.

**Figure 2.**
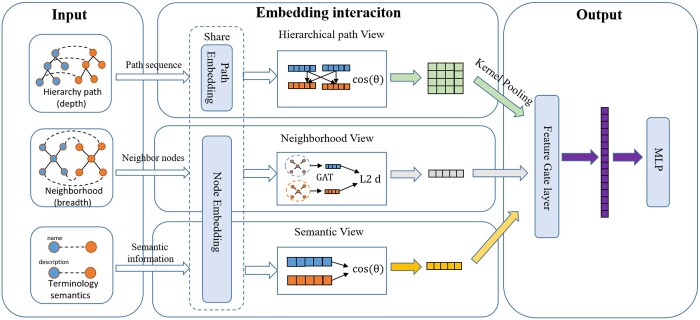
This diagram illustrates the proposed method for comprehensive terminology alignment, which consists of three views: semantic view, neighborhood view, and path view. Each view’s input is fed into a shared BERT model to obtain corresponding feature representations. The feature vectors are then interacted and calculated using a distance function. Finally, the resulting scores are obtained through an MLP with a gate mechanism.

Framework. In order to fully leverage the hierarchical and neighborhood information present in the terminology base, we propose a multi-view framework that considers the semantic, neighborhood, and hierarchical features of terms. As shown in Fig. 2, our framework is composed of three components: (i) The semantic view generates embedding for term’s name using BERT-based model and computes semantic scores based on similarity scores. (ii) The neighborhood view component leverages a Graph Attention Network model to aggregate the neighborhood scores for each term. (iii) The path view concatenates the hierarchical structure into a context and use BERT-based model to calculate similarity matrices, which are subsequently aggregated to obtain path scores.

Finally, the output model assigns different weights to the different scores and produces the final ranking score. It is worth noting that the approach used in this study did not separately consider the influence of attributes on terminology, due to the significant differences in terminology attributes across various systems.

## 3 Materials and methods

In this section, we will begin by introducing the interaction methods used for the three views we adopted. Next, we will describe our approach for feature fusion. Finally, we introduce the details of our training method.

### 3.1 Multi-view interaction

In the multi-view interaction module, we learn representations of the terminology from multiple views, including semantic, neighborhood, and hierarchical path. We then compute the interaction scores between these views to combine their complementary information.

**View 1 (Semantic information).** Semantic information refers to the name or description of a biomedical concept. In our study, we utilize the PubMedBERT (the Hugging Face URL https://huggingface.co/microsoft/BiomedNLP-PubMedBERT-base-uncased-abstract), a biomedical model trained on abstracts from PubMed, to process its name as input and extract its semantic information. Specifically, we use the 〈CLS〉 embedding to represent the term’s name, as follows:


(1) $$V(e)=\text{MLP}({\text{BERT}}_{\mathrm{CLS}}(e)),$$

where V(e) represents the embedding vector of the term and MLP is a fully connected layer that is used to transform the output vector into a specified dimension. After obtaining the semantic vectors, we calculate similarity between two terms’ vectors using cosine similarity, as shown below:


(2) $$\text{sim}(e,e\prime )=\frac{V(e)\cdot V(e\prime )}{\left|V(e)|\cdot |V(e\prime )\right|}.$$

Here, V(e) and V(e′) denote the vector of concept’s name, and sim(e,e′) represents the semantic interaction score.

**View 2 (Neighborhood information).** The neighborhood information of a term is considered as a set of adjacent nodes. GNN models have significant advantages in aggregating such neighborhood information. In this study, we adopt the popular GAT model to weight the neighborhood information using attention mechanisms which can better incorporate node features. The core formula for this weighting is expressed as follows:


(3) $$h\prime_{i}=\sigma \left(\frac{1}{K}\sum_{k=1}^{K}\sum_{j\in N_{i}}\alpha_{ij}^{k}W^{k}h_{j}\right)$$

(4) $$\alpha_{ij}=\text{softmax}(c_{ij})=\frac{\;\text{exp}(c_{ij})}{\sum_{k\in N_{i}}\;\text{exp}\;(c_{ik})}$$

(5) $$c_{ij}=\text{LeakyReLU}(q^{⊺}[Wh_{i}⊕Wh_{j}]),$$

where the symbol $h\prime_{i}$ denotes the output vector of the current GAT layer, $\alpha_{ij}^{k}$ represents the normalized attention coefficient computed by the *k*th attention mechanism, and $W^{k}$ denotes the weight matrix of the corresponding input linear transformation. LeakyReLU is a nonlinear function, *q* is a learnable parameter.

Finally, we compute the neighborhood interaction score between *e* and e′ for this view by utilizing the *l*_2_ distance to aggregate information from different neighborhoods through GAT.

**View 3 (Hierarchical path information).** A hierarchical path is defined as a sequence of connected edges between nodes at different levels of a hierarchical structure, such as a tree or a taxonomy. To capture long-term dependencies within hierarchical structures, we adopt a holistic approach to represent hierarchical paths using BERT-based models. Specifically, we partition the hierarchical path into three segments: ancestor nodes, current node, and child nodes, with the number of ancestor and child nodes controlled by parameters *n* and *k*, respectively. Notably, the top-down construction of the path ensures a consistent and meaningful representation.

We concatenate these three sequence parts with a separator 〈SEP〉 and use the resulting sequence as input to BERT. The following equation illustrates how we construct the path sequence:


(6) $$P(e)=\{\langle \mathrm{CLS}\rangle ,e^{\mathrm{anc}},\langle \mathrm{SEP}\rangle ,e,\langle \mathrm{SEP}\rangle ,e^{\mathrm{child}},\langle \mathrm{SEP}\rangle \}$$

(7) $$e^{\mathrm{anc}}={\left\{e_{i+1}|(e_{i},h_{i},e_{i+1}),e_{1}=e\right\}}_{i=1}^{\left|n-k\right|}$$

(8) $$e^{\mathrm{child}}={\left\{e_{j}|(e_{j},h_{j},e_{j+1}),e_{k}=e\right\}}_{j=1}^{\left|k-1\right|},$$

Here, $e^{\mathrm{anc}}$ and $e^{\mathrm{child}}$ represent the ancestor and child nodes, respectively, and 〈SEP〉  is the separator used to concatenate the three parts of the hierarchical path. *P*(*e*) is the final path sequence that is inputted into BERT. The parameter n represents the length of the hierarchical path, and k represents the position of the aligned node. For example, if the path length *n* is 5 and *k* is 2, this indicates that there are three ancestor nodes and one child node.

In the path interaction module, we aim to calculate the hierarchical differences between different entities in order to filter out entities with similar hierarchical paths. Since each node may have multiple hierarchical paths, we consider using a similarity matrix to represent the scores between different paths. Formally, given two path sets N(P(e)) and N(P(e′)), we calculate the cosine similarity matrix ***S***, as follows:


(9) $$S_{i,j}=\frac{V(P(e_{i}))\cdot V(P(e\prime_{j}))}{\left|V(P(e_{i})|\cdot |V(P(e\prime_{j}))\right|},$$

where $S_{i,j}$ represents the cosine similarity between $P(e_{i})$ and $P(e\prime_{j})$.

Finally, we aim to aggregate the similarity matrix of the overall path’s 〈CLS〉 vector using kernel pooling (Xiong *et al.* 2017). We employ a *k*-dimensional RBF kernel function to map the similarities of each row and column of the matrix and aggregate them. The resulting interaction score of the hierarchical path is given by:


(10) $$K_{k}(S_{i})=\sum_{j=1}^{\left|N(e\prime )\right|}\;\text{exp}\;\left(-\frac{{\left(S_{i,j}-\mu_{k}\right)}^{2}}{2\sigma_{k}^{2}}\right)$$

(11) $$K^{\mathrm{row}}(S_{i})=\{K_{1}(S_{i}),\ldots ,K_{K}(S_{i})\}$$

(12) $$\mathrm{Agg}(S)=\sum_{i=1}^{\left|N(e)\right|}\;\text{log}\;(K^{\mathrm{row}}(S_{i}))⊕\sum_{j=1}^{\left|N(e\prime )\right|}\;\text{log}\;(K^{\mathrm{col}}(S_{j}))$$

The similarity score of the similarity matrix is represented by *Agg*(*S*), which is obtained by concatenating $S_{\mathrm{row}}$ and $S_{\mathrm{col}}$ to obtain the final similarity score. By combining it with the neighborhood information, we can simultaneously learn multi-level information of both the neighborhood and the path.

### 3.2 Feature fusion

To model multi-view information for medical terminology, we have computed three types of interaction scores for each pair of entities (*e*, e′). We use the feature gate layer to select the most salient latent information at the feature level (Huang *et al.* 2020).

For each feature interaction vector, denoted as ***h***, we calculate the gate value that represents the importance of the embedding at the feature level. The gate value is obtained by passing the feature embedding through a nonlinear activation function *σ*, followed by a learned parameter matrix $W_{i}$:


(13) $$g_{i}=\sigma (W_{i}\cdot h_{i}).$$

We then assign the gate value to the corresponding feature embedding to generate a gate-aware embedding:


(14) $$h\prime_{i}=g_{i}⊙h_{i}.$$

Here, the symbol ⊙ represents element-wise multiplication. The feature embedding vector is denoted as $h_{i}\in R^{k}$, where *k* is the dimensionality of the *i*th original embedding. For this study, *i* takes on the values 1, 2, and 3.

We collect all feature gating embedding and concatenate them as input to the MLP module, which ultimately yields the alignment score between entities:


(15) $$\mathrm{score}(e,e\prime )=\text{MLP}([h\prime_{1},h\prime_{2},h\prime_{3}]).$$

By incorporating feature embedding gates, we can effectively select and emphasize the most informative features, which helps to improve the model’s performance and interpretability.

### 3.3 Training method

To improve the learning of medical terminologies, we utilize the PubMedBert and fine-tuned it using prealigned seeds. For training the learning-to-rank model, we use the pairwise margin loss function due to its effectiveness (Pahikkala *et al.* 2007). The loss function is defined as follows:


(16) $$L_{\mathrm{pair}}=\sum_{(e,e\prime^{+},e\prime^{-})\in E}max\{0,\gamma +d(e,e\prime^{+})-d(e,e\prime^{-})\},$$

where *γ* is the margin enforced between positive and negative pairs. *e* and e′ are terms from different sets, i.e. e∈E and e′∈E′. $d(e,e\prime^{+})$ and $d(e,e\prime^{-})$ denote the *l*_1_ distances between a term *e* and its corresponding positive and negative samples, respectively.

To construct negative examples, we use a combination of random sampling and the *k*-nearest neighbors method. For each positive example $\left(e,e\prime^{+}\right)$, we generate five negative examples relevant to the positive examples using the *k*-nearest neighbors method and introduce some degree of randomness to the sampling process (Robinson *et al.* 2021). Specifically, we first generate rough entity embedding for *G* and G′. Then, we select the 50 entities closest to *e* in the rough embedding space, and the negative sample are randomly selected from these 50 pairs.

## 4 Experiments

In this section, we first compare our proposed approach with various baseline methods. Subsequently, we conduct an ablation study to further analyze our model’s performance. Finally, we conduct a detailed analysis to examine the impact of different pretraining models and training proportions. Additionally, we extend our experiments to include the ontological dataset.

### 4.1 Experiment setup

**Datasets.** To evaluate the effectiveness of our proposed method in aligning medical terminologies, we analyzed publicly available mapping files in the biomedical domain, including ICD10-ICD11(downloaded from https://icd.who.int/browse11/l-m/en), ICD9CM3-SNOMED, and SNOMED-ICD10 (downloaded from https://www.nlm.nih.gov/healthit). The ICD10-ICD11 and SNOMED-ICD10 are mapping sets that related to diseases, while ICD9CM3-SNOMED is a mapping set related to surgeries. The architectural structures of ICD10 and ICD11 are relatively similar, while the differences between SNOMED-CT and ICD10 are significant. By analyzing these three datasets, we can effectively verify the efficacy of our proposed method in term alignment within different medical terminology classifications and under varying architectural structures. Table 1 outlines the detail information of our three datasets.

**Table 1.** This table describes statistics of ICD10-ICD11, ICD9CM3-SNOMED, and SNOMED-ICD10.

No.	Dataset	Entity scale	Relation scale	Seeds
0	ICD10-ICD11	(11 532, 26 411)	(11 527, 26 381)	10 461
1	ICD9CM3-SNOMED	(4650, 71 585)	(4551, 243 208)	1734
2	SNOMED-ICD10	(108 253, 11 532)	(354 434, 11 527)	14 274

**Baseline methods.** We compare two categories of entity alignment methods: the GCN-based methods and the BERT-based methods. The GCN-based methods, such as HGCN (Gao *et al.* 2022) and RDGCN (Wu *et al.* 2019), while effective at capturing local graph structures, may be have limitations in capturing more complex information between entities. In contrast, the BERT-based methods, represented by HMAN (Yang *et al.* 2019) and Bert-Int (Tang *et al.* 2020), utilize pretrained models for embedding and incorporate multiple views of information, including neighborhood and semantic aspects, to achieve accurate entity alignment. Our comparison of these two categories of methods provides important insights into the strengths and limitations of each approach.

**Implementation details**. In our study, we select 30% of the aligned seeds as the training set and the remaining 70% for validation. During the specific processing, we preselected SNOMED-CT data based on the classification of data (diseases, surgeries) to reduce the range of data candidates. Furthermore, in order to improve training efficiency and control memory usage, we limit the seed alignment within 15 000. The dimension of the BERT embedding is 768. We use a 300D MLP in Equation (1) and both GCN models used in this study were constructed using two layers.

**Metrics.** The performance of our model is evaluate utilizing Hits@1 and Hits@10, widely recognized metrics for entity alignment tasks, where Hits@k denotes the proportion of accurately aligned entities among the top *k* ranked entities.

### 4.2 Overall evaluation

We compare our proposed method with all mainstream entity alignment methods on the test set, and the results are presented in Table 2.

**Table 2.** Evaluation results on the datasets.

Methods	ICD10-ICD11		ICD9-SNOMED		SNOMED-ICD10	
	Hits@1	Hits@10	Hits@1	Hits@10	Hits@1	Hits@10
Ours model	0.726	0.918	0.884	0.991	0.422	0.758
Methods that solely rely on graph structure						
GCNAlign	0.213	0.449	0.249	0.575	0.237	0.532
RDGCN	0.232	0.463	0.245	0.579	0.239	0.535
HGCN	0.236	0.467	0.246	0.583	0.241	0.537
Methods that combine pretraining language models						
BERT	0.657	0.894	0.882	0.978	0.399	0.728
BERT+GCN	0.661	0.890	0.879	0.983	0.402	0.735
HMAN	0.687	0.901	0.883	0.984	0.403	0.741
BERT-Int	0.710	0.909	0.881	0.987	0.407	0.751

After analyzing the results presented in Table 2, we conclude the following conclusions: (i) The incorporation of pretrained models into graph neural networks potentially yield superior performance compared to utilizing graph neural networks in isolation. (ii) Our proposed model, which incorporates path information, outperforms other baseline models in terms of Hit@1 and Hit@10. Specifically, our method achieved about 2% improvement over the previous state-of-the-art BERT-based method, BERT-Int, on the ICD10-ICD11 dataset. This highlights the importance of considering the hierarchical structure of medical terminology in achieving accurate alignment. The poor performance of HMAN and BERT-Int may be attributed to their limited consideration of the heterogeneity of neighborhood structures and semantic features in different medical terminology systems, which can result in alignment errors. In contrast, our approach takes into account multiple views, including path, neighborhood, and semantic features, which allows for a more comprehensive understanding of medical terminology alignment.

### 4.3 Ablation study

To further investigate the importance of each block in our framework, we conducted an ablation study, which is presented in Table 3. Specifically, we conducted two sets of experiment, as follows:

**Table 3.** Ablation analysis on the datasets.

Models	ICD10-ICD11		ICD9-SNOMED		SNOMED-ICD10	
	Hits@1	Hits@10	Hits@1	Hits@10	Hits@1	Hits@10
Ours model	0.726	0.918	0.884	0.991	0.422	0.758
Remove different views of method						
Semantic	0.555	0.905	0.457	0.951	0.325	0.707
Neighbors	0.719	0.917	0.885	0.983	0.419	0.753
Path	0.686	0.897	0.879	0.985	0.405	0.747
Neighbors and path	0.657	0.894	0.882	0.978	0.399	0.728
Neighbors and semantic	0.629	0.895	0.504	0.933	0.324	0.701
Path and semantic	0.522	0.835	0.286	0.843	0.273	0.616
Replace certain method in the specific view						
Remove Kernel pooling	0.722	0.918	0.880	0.989	0.417	0.753
Remove Gate-layer	0.723	0.918	0.883	0.989	0.419	0.754
GAT → GCN	0.717	0.916	0.882	0.986	0.418	0.747
Add max pooling	0.725	0.917	0.883	0.990	0.419	0.755

**Remove different views of our method.** To evaluate the impact of different views on the performance of our model, we remove each view one by one to assess their contributions to the overall score. In addition, we also analyze the performance of our model by only retaining a single view module to observe the changes in model performance under a single view.

The results of our study indicate that removing the path view leads to a 4% decrease in performance, while removing the neighborhood view caused only a 0.7% decrease in the ICD10-ICD11 dataset. This suggests that in the context of terminology alignment, hierarchical path information may be more important than neighborhood information. Moreover, removing both neighborhood and path views results in a significant decrease in model performance, particularly in the ICD-10 to ICD-11 alignment dataset, indicating that structural features play a critical role in achieving accurate entity alignment in medical terminologies. Furthermore, when only retaining the semantic, neighborhood, and path views, we find that the semantic view has the greatest impact on the overall alignment score, while the neighborhood view has the least impact. The path view occupies an intermediate position between these two extremes. These findings suggest that relying solely on neighborhood information may not be sufficient for precise alignment and emphasize the importance of considering the full range of available information.

**Replace certain method in the specific view.** To assess the effectiveness of our proposed method in different views, we conduct ablation experiments by removing or replacing specific computational components.

The results show that when the RBF kernel pooling module in the path-view is removed, the model’s performance decreased by 0.4%. Similarly, removing the gate layer in the final output model led to a performance decrease of up to 0.3%. Furthermore, we explore the possibility of replacing the GAT model with a GCN model, but this results in a larger performance decrease of 0.9%. While these effects may not be significant, these results demonstrate the contributions of these components to the overall performance of the model.

### 4.4 Detailed analysis

In this section, we explore the impact of more specific factors on the performance of our proposed model for biomedical alignment tasks, as follows:

**The impact of different pretraining language model.** To compare the impact of different pretrained language models, we attempt to replace various pretrained language models and analyze the differences among them.

As shown in Table 4, our experimental results indicate that the pretrained language model trained on general-purpose corpora, such as BERT, Roberta (Liu *et al.* 2020), demonstrate relatively inferior performance compared to models that are specifically pretrained on the biomedical data, such as Pub-MedBERT (Xie *et al.* 2022) and BioBERT (Lee *et al.* 2020). Notably, PubMedBERT, trained on abstracts, achieves the highest performance, surpassing the foundational BERT model by over 10%. These findings underscore the critical importance of incorporating biomedical domain knowledge into alignment models. When aiming for optimal performance, it is essential to consider the specific domain of the task when selecting pretrained language models.

**Table 4.** Results of using different pretraining language models.

Models	ICD10-ICD11		ICD9-SNOMED		SNOMED-ICD10	
	Hits@1	Hits@10	Hits@1	Hits@10	Hits@1	Hits@10
Change the pretraining language model						
PubMedBERT	0.726	0.918	0.884	0.991	0.422	0.758
BERT	0.604	0.821	0.817	0.937	0.252	0.524
BERT-multi	0.644	0.853	0.831	0.947	0.236	0.525
RoBERTa	0.605	0.856	0.765	0.923	0.206	0.513
BioBERT-v1.1	0.714	0.915	0.867	0.978	0.392	0.732

**Figure 3.**
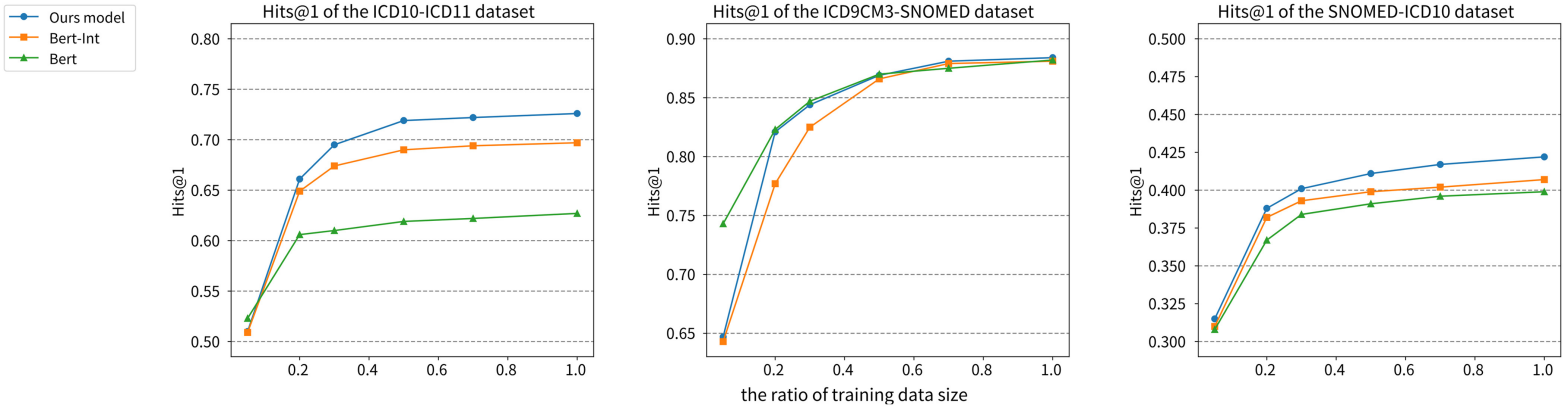
Performance comparison of BERT, BERT-Int, and our model across different training data sizes, measured by Hits@1, at 5%, 10%, 20%, 50%, and 70% Training Data Proportions.

**The impact of different sizes of training data.** To study how the size of training set influences the performance and to evaluate the scalability of the proposed model, we use different proportions of the training data and calculate the Hits@1 score. Considering the total number of training data and the cost of time, we pick 5%, 10%, 20%, 50%, 70%, and all of the training data. We compare the results of our proposed method with those of BERT and BERT-Int at different training proportions, and the comparative results are presented in Fig. 3.

As shown in Fig. 3, we observe that the three BERT-based models achieve hits@1 scores that are very close to the performance of the full training data when trained on only 50%–60% of the data, and further increasing the size of the training set only lead to the limited performance improvement. It seems that the model may have already learned the important patterns and features from the available data, and adding more data may not provide much additional information. Moreover, by comparing our proposed method with BERT and BERT-Int, we find that their performance differences are small in the case of a small-sized training set. However, as the size of the training set increased, our model’s performance improvement surpassed the other methods. This may indicate that our model learns to combine hierarchical path information during the training process, which allows it to achieve better performance.

### 4.5 Assessing the extension of method for ontology

We extend and evaluate our method using the Ontology dataset provided by BioPortal to show the potential of our method for the alignment of redundant ontologies. Specifically, we utilize the COVID-19 Ontology and Gene Ontology (GO) mappings, and employee synonym replacements for the names (50%) in COVID and GO ontologies to simulate scenarios where the semantic names of entities differ.

As shown in Table 5, the experimental results demonstrate that our method remains effective in the ontology mapping between COVID and GO. In particular, our method demonstrates superior performance compared to other baseline methods when employing synonym replacements for terminologies in COVID and GO. This finding indicates that our approach is more effective in scenarios involving semantic ambiguity. It is worth noting that not all contextual information proves helpful, as the BERT-Int and GCN methods do not exhibit significant performance improvements in cases of semantic ambiguity. This suggests that classification paths may offer greater advantages in alignment compared to the neighborhood structure for ontology.

**Table 5.** Results of methods in mapping COVID ontology to gene ontology.

Models	COVID-GO		COVID(syn)-GO		COVID-GO(syn)	
	Hits@1	Hits@10	Hits@1	Hits@10	Hits@1	Hits@10
Ours model	0.976	0.984	0.885	0.957	0.857	0.956
BERT	0.973	0.985	0.874	0.955	0.845	0.956
BERT+GCN	0.954	0.986	0.840	0.954	0.830	0.957
BERT-Int	0.968	0.985	0.849	0.960	0.841	0.958

## 5 Conclusion and future work

In this paper, we propose a multi-view alignment method that leverages the hierarchical structure to establish associations between the different medical terminology bases. We conduct experimental evaluations on publicly available medical terminology mapping datasets and extend our method to include otology data in BioPortal. The results confirm the effectiveness of our proposed method, with improvements observed in Hits@1 and Hits@10 compared to baseline methods.

In future work, the alignment information obtained from multiple medical terminology bases can be applied to downstream applications, such as improving biomedical translations and promoting terminology standardization in the medical field (Luo *et al.* 2019, 2022). This can help improve the accuracy, efficiency, and interoperability of medical data exchange across different healthcare systems and organizations. Moreover, the alignment information between different terminology databases could serve as pretraining data for large-scale generative models trained on biomedical corpora, which could further advance natural language processing in the medical domain (Michalopoulos *et al.* 2021).
